# XUV Absorption Spectroscopy and Photoconversion of a Tin-Oxo Cage Photoresist

**DOI:** 10.1021/acs.jpcc.3c07480

**Published:** 2024-02-27

**Authors:** Najmeh Sadegh, Quentin Evrard, Peter M. Kraus, Albert M. Brouwer

**Affiliations:** † Advanced Research Center for Nanolithography, Science Park 106, 1098 XG Amsterdam, The Netherlands; ‡ Department of Physics and Astronomy, and LaserLaB, Vrije Universiteit, De Boelelaan 1105, 1081 HV Amsterdam, The Netherlands; § van ‘t Hoff Institute for Molecular Sciences, University of Amsterdam, Science Park 904, 1098 XH Amsterdam, The Netherlands

## Abstract

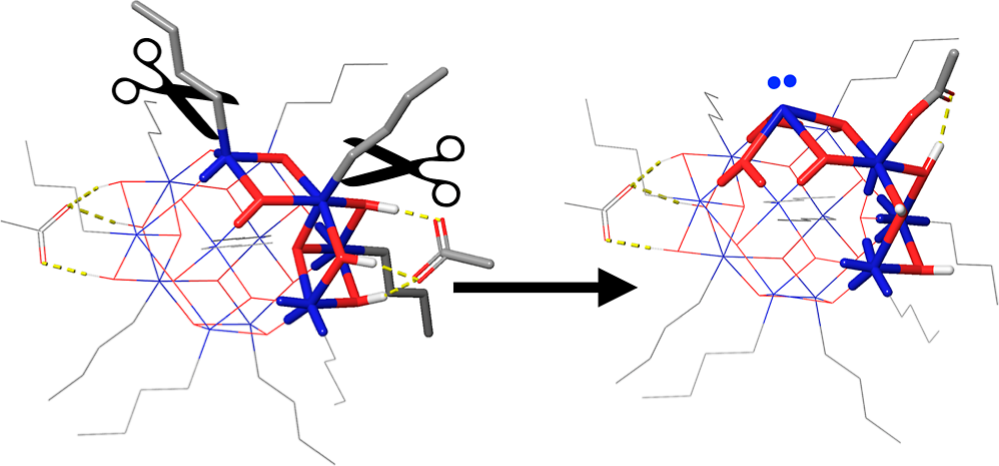

Extreme ultraviolet lithography has recently been introduced in high-volume production of integrated circuits for manufacturing the smallest features in high-end computer chips. Hybrid organic/inorganic materials are considered as the next generation of photoresists for this technology, but detailed knowledge about the response of such materials to the ionizing radiation used (13.5 nm, 92 eV) is still scarce. In the present work, we use broadband high-harmonic radiation in the energy range 22–70 eV for absorption spectroscopy and photobleaching (that is, the decrease of absorbance) of thin films of an *n*-butyltin oxo-cage, a representative of the class of metal-based EUV photoresist. The shape of the absorption spectrum in the range 22–92 eV matches well with the spectrum predicted using tabulated atomic cross sections. The photobleaching results are consistent with loss of the butyl side groups due to the breaking of Sn–C bonds following photoionization. Bleaching is strongest in the low-energy range (<40 eV), where the absorption is largely due to the carbon atoms in the organic groups. At higher energies (42–70 eV), absorption is dominated by the tin atoms, and since these remain in the film after photoconversion, the absorption change in this region is smaller. It is estimated that after prolonged irradiation (up to ∼3 J cm^–2^ in the range 22–40 eV) about 70% of the hydrocarbon groups are removed from the film. The rate of bleaching is high at the beginning of exposure, but it rapidly decreases with increasing conversion. We rationalize this using density functional theory calculations: the first Sn–C bonds are efficiently cleaved (quantum yield Φ ≈ 0.9), because the highest occupied molecular orbitals (HOMOs) (from which an electron is removed after photoionization) are located on Sn–C sigma bonds. In the photoproducts, the HOMO is localized on tin atoms that have lost their hydrocarbon group (formally reduced to the Sn(II) oxidation state), and holes formed on those tin atoms lead to less efficient cleavage reactions. Our results reveal the primary reaction steps following excitation with ionizing radiation of tin-oxo cages. Our methodology represents a systematic approach of studying and quantitatively assessing the performance of new photoresists and as such enables the development of future EUV photoresists.

## Introduction

Extreme ultraviolet lithography is taking up a preeminent role in the semiconductor industry for producing the smallest features in integrated circuits, as it pushes the diffraction limit to <10 nm by using photons with a wavelength of 13.5 nm.^1,2^ Enablers for continuing feature shrinkage are better lithography scanners, for example, with higher numerical apertures, the market introduction of which has already been announced. However, the current photoresists for EUV lithography are adapted from those developed for 193-nm immersion lithography and these will soon become the limiting factor for continuing shrinkage in lithography and thus microelectronic fabrication.^3,4^ These currently used resists are based on organic polymers, of which the solubility is switched from organic solvent-soluble to aqueous base-soluble by acid-catalyzed removal of dissolution-inhibiting groups. The acid is locally photogenerated in the film areas to be dissolved in the developer. Because a single acid equivalent generated can cause many deprotecting reactions, this type of resist is known as chemically amplified resist (CAR).^5^ This amplification mechanism wanted for 193 nm immersion lithography is now becoming exactly what is limiting very high resolution work in EUV lithography: the diffusion associated with the amplification mechanism is undesirable, as it leads to blurring. Therefore, in the EUV application it is drastically reduced by adding base quencher, taking away the advantage in photon economy.^6^ Other disadvantages of organic-based materials are that their absorption cross sections at the EUV wavelength are small,^7−9^ and their etch resistance in thin films is low.^10^ For these reasons, research has been initiated in hybrid organic/inorganic molecular materials, which combine strong absorption, high etch resistance, and small size (not limiting resolution).^11−25^

For the photoresists used in lithography the step from ultraviolet (193 nm) to EUV implies a very different mechanism of activation.^26−29^ UV photons cause electronic excitation to abound state of a specific chromophore (the photoacid generator in CAR), from which the primary reaction occurs. EUV photons (energy 92 eV) on the other hand cause ionization of all resist components, depending on atomic cross sections. The primary photoelectrons mostly lose their kinetic energy by inelastic scattering, releasing more electrons and holes and possibly generating electronically and vibrationally excited states. It is commonly thought that the secondary electrons play a key role in the chemical reactivity of photoresists in the EUV regime.^26,30^ To understand the mechanisms of energy loss and reactions, the electronic states in the energy range *E*_photon_ < 92 eV have to be characterized. When the photon energy is decreased from 92 eV down to the ionization threshold, ionization can still occur, but with lower and lower yields of secondary electrons. By studying the electron yield and the photochemical conversion in the XUV regime, the role of secondary electrons can be investigated.

The present paper focuses on the absorption and photoconversion in the XUV spectral range (22–70 eV) of a tin-oxo cage photoresist model. A preliminary study on electron generation in the same energy range has recently been published.^31^ In the previous and present work, we utilized a tabletop laser-driven extreme-ultraviolet source based on high-harmonic generation, which allows broadband photobleaching studies of EUV photoresist. Tin-oxo cages were introduced in the open literature on EUV photoresists by Brainard and coworkers.^14^ The compounds were shown to behave as negative tone resists, which means that the irradiated part of the thin film becomes insoluble in a developer. Tin–carbon bond cleavage was proposed as the primary reaction. The subsequent coupling between neighboring cages was suggested to be the process that leads to the loss of solubility.^14,32,33^ Our group has shown similar types of reactions upon exposure to deep UV and to electrons.^34−38^

**Chart 1:**
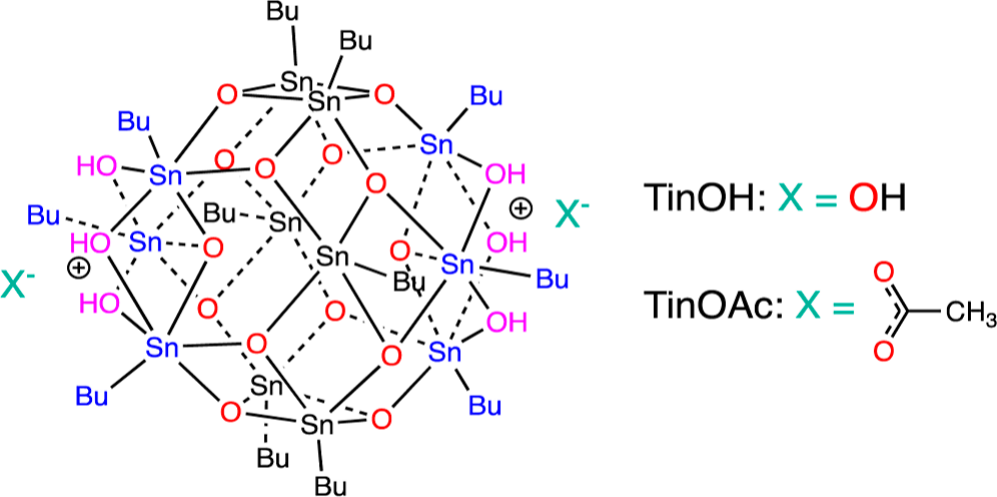
Chemical Structures of *n*-Butyltin-Oxo Cages with Hydroxide and Acetate Counterionsa

The chemical structure of the compound investigated in the present study, named TinOAc, is shown in Chart 1. In a preliminary study on the related TinOH, we measured the absorption spectrum and photobleaching in a smaller energy range (25–40 eV).^39^ In the present work, we give a full account of our experiments with an improved setup, covering a larger energy range and a quantitative evaluation that allows us to estimate the quantum yield of tin–carbon bond breaking. Quantum chemical calculations are used to identify the likely reaction pathways.

a SnBu units colored blue are 6-coordinated Sn, in the cap regions of the molecule, where the bridging OH groups are highlighted in pink color. SnBu in black are part of the central belt.

## Experimental Section

The experimental setup used in the present work has been recently described.^40^ Briefly, higher harmonics of an 800 nm pump laser running at 2 kHz are generated in a finite gas cell with argon (22–42 eV) or with neon (42–70 eV). By combining the 800 nm source with the frequency doubled 400 nm output, odd and even harmonics are produced in the case of high-harmonic generation from argon, to increase the spectral coverage.^41,42^ The spectra of the irradiance at the sample in the two energy ranges are shown in Figure S1 (Supporting Information).

These two harmonic spectra were used as the source of exposure for performing absorption and photobleaching measurements separately in the low-energy (22–42 eV) and high-energy (42–70 eV) ranges. The argon and neon backing pressures were 20 and 36 mbar, respectively. The numbers of photons on the sample correspond to 0.2 nJ per pulse in the low-energy region and 4.5 pJ per pulse in the high-energy range. The conversion efficiency in the generation process of harmonics is clearly lower in Ne than in Ar. The exposed area was measured to be 1.2 × 10^–4^ cm^2^ giving a power density of ∼3.3 mW cm^–2^ in the low energy range. In the high-energy range, the power density was ∼0.1 mW cm^–2^.

Samples of TinOAc were spin coated from toluene solutions on silicon nitride membranes (3 × 3 mm^2^ windows, 30 nm thick, on 7.5 × 7.5 mm^2^ Si frames) purchased from Norcada.^35,43^ We used different samples for the experiments in the two different energy ranges. The typical thickness of the TinOAc films is 30–50 nm,^35^ but there is some variation from sample to sample, and the thicknesses of the films on the fragile SiN membranes could not be directly measured by independent methods. Absorption spectra [*A* = −ln(*I*_s_/*I*_mem_)] are obtained by alternately measuring the transmission of the XUV beams through the sample (*I*_s_) and a blank SiN reference (*I*_mem_), each during a fixed time interval in which 20 acquisitions of the camera were averaged. In the low energy range, the exposure time was 55 ms; in the high-energy range, it was 200 ms. The sample was exposed 17 ms longer due to the open time of the shutter that blocks the beam between acquisitions. Absorbances are calculated at the peaks (peak maximum ±5 data points) of the HHG spectra. The intensities at energies between the peaks are too low to obtain reliable data. During the experiment, the absorption spectra gradually change due to photobleaching, but especially in the high-energy range the spectra *A*(λ,*t*) show considerable fluctuations due to random measurement error. Therefore, the calculated absorbances were smoothed by fitting the time traces *A*_λ_(*t*) at each of the HHG peaks to an exponential or biexponential decay curve. This allows us to derive the spectra at the start and end of exposure making use of the entire data set and to monitor the photobleaching process.

The exposure dose at the sample was estimated based on data provided by the manufacturers of the grating and the CCD camera, transmission values of the Al filter assuming a 4-nm oxide layer on both sides, and the measured transmission of the silicon nitride membrane, as outlined in ref (40). An independent calibration was not possible, but we consider the calculated photon fluxes at the sample to be reliable to ±50%. The overall pulse energies derived from the data agree with published data from similar setups.^40,44,45^ During the measurement, the irradiance was monitored using the measurements through the membrane, and the accumulated dose on the sample was corrected for drift and fluctuations. For the experiments with HHG generation in neon, we could monitor only the high-energy range, so the estimation of the power is more approximate.

Using the photon fluxes on the sample and the measured transmission, we calculated the number of absorbed photons and the absorption change per time interval, allowing us to estimate the photochemical quantum yield as discussed below.

To support the discussion of possible reaction mechanisms, quantum chemical calculations were performed on the complex of the oxo cage dication with two acetate anions and on a number of potential reaction products. We used the B3LYP hybrid functional with the Def2SVP effective core potential basis set for geometry optimization and with the larger Def2TZVP basis set for single-point energy evaluations. To mimic the effect of the dielectric medium provided by the surrounding molecules in the thin films, we used the polarizable continuum model (PCM) with parameters for diethyl ether. The solid state has a low polarity compared with solvents because of its limited ability to rearrange its structure. On the other hand, the tin-oxo cage complexes are multipolar species, so we considered that a moderately polar solvent (dielectric constant 4.2) should be a reasonable mimic to test the effect of environment on the relative energies.^46^ Optimization of structures with the PCM showed only minor changes in geometries and relative energies, so the numbers reported are all single-point energies at the optimized geometries of the isolated molecules. The Gaussian16 program package was used.^47^

## Results

### XUV Absorption Spectra

**Figure 1:**
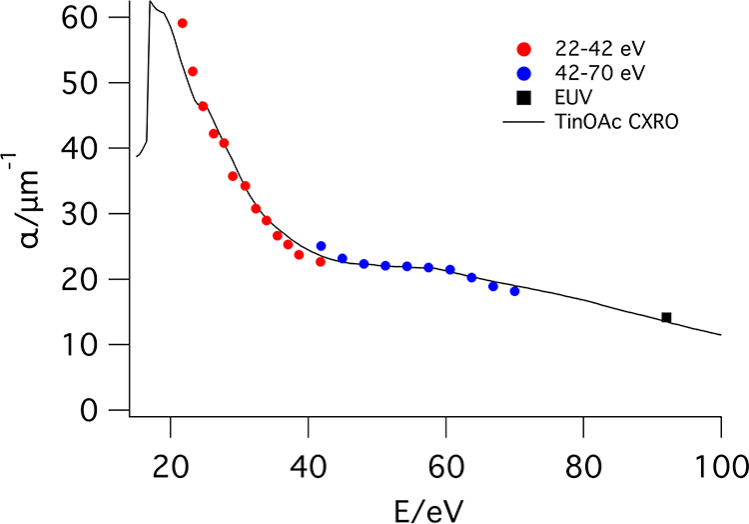
Comparison between the predicted and the measured XUV absorption spectra of TinOAc. The data point at 92 eV (black square) is from ref (49).

Figure 1 shows the experimentally determined absorption spectrum of the TinOAc films together with the predicted one calculated from the atomic scattering factors from the CXRO database (see the Supporting Information for details).^48^ The spectra are expressed in terms of the absorption factor α = *A*/*z*, where *A* is the absorbance and *z* the film thickness. The spectrum over the range 22–70 eV is constructed from the absorption data from separate measurements in the low- and high-energy ranges performed on two different TinOAc samples. Because thickness measurements of films on thin membranes are challenging, we validate the thickness in the following way: for both energy ranges, the measured absorption spectra are fitted with a single constant to the predicted absorption spectrum. The film thickness corresponding to the best fit of experimental to predicted spectra is 46 nm for the sample used in the low energy range and 51 nm for the one in the high-energy range (see the Supporting Information for more details). These numbers are close to the expected film thickness measured by using the same spin coating conditions on silicon substrates. An additional data point shown at 92 eV stems from our earlier work, in which we determined an absorption factor α = 14.2 μm^–1^.^49^ Overall, there is very good agreement between the shape of the measured absorption spectrum and the predicted one.

### XUV-Induced Bleaching

**Figure 2:**
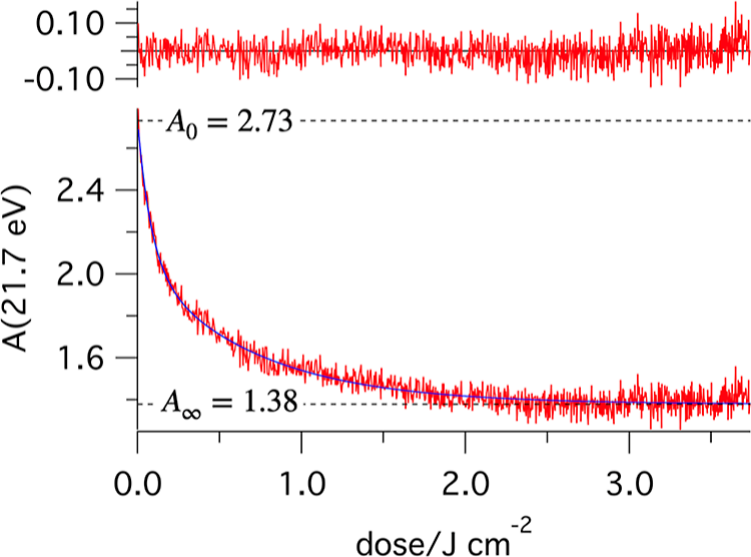
Example of a time trace of absorbance (measured at 21.7 eV), fitted with a biexponential decay. Residuals are shown at the top. The fitted values at *t* = 0 and *t* = ∞ are indicated with dashed lines. The same approach was used to determine the absorbance values for the other HHG peak energies.

The absorption spectrum of TinOAc plotted in Figure 1 is constructed by extrapolation of the recorded spectra to the start of the exposure. Curves of absorbance versus the total XUV dose impinging on the sample are shown in Figures S2 and S3 (Supporting Information). A representative example is given in Figure 2.

**Figure 3:**
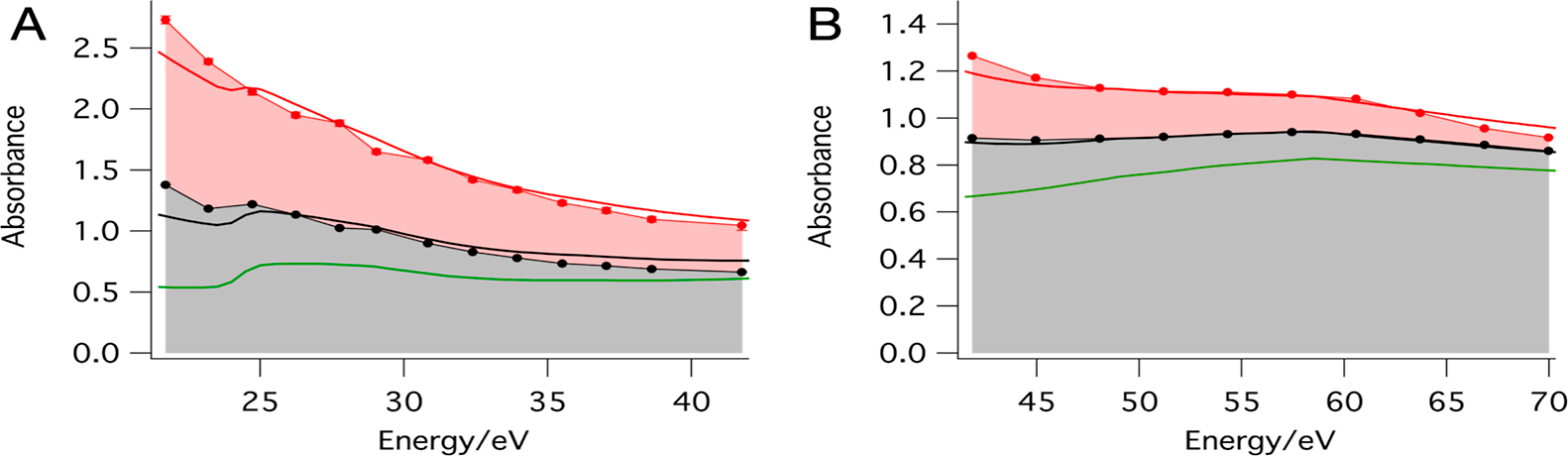
Absorption spectra of TinOAc thin films extrapolated to *t* = 0 (*A*_0_^obs^, red markers) and to *t* = ∞ (*A*_∞_^obs^, black markers). The solid red line is the CXRO predicted spectrum for TinOAc (*A*_0_^pred^), and the green line is that of Sn_12_O_18_, with the same surface density (*A*_∞_^pred^). The spectra *A*_∞_ are fitted to a linear combination of *A*_0_^pred^ and *A*_∞_^pred^ according to eq 3 and represented with the black lines. The red area corresponds to the bleachable part of the absorption, and the gray area corresponds to the unbleachable part. (A) In the range of 22–42 eV, film thickness of 46.2 ± 2.4 nm. (B) In the range of 42–70 eV, thickness 50.5 ± 1.1 nm.

To visualize the induced changes in the spectrum of the resist with exposure, the spectra at *t* = 0 and *t* = ∞ are plotted in Figure 3 for the measurements in the low XUV energy range and the higher energy range separately. In the low energy range (Figure 3A) the sample is exposed to up to ∼3.6 J cm^–2^ and we clearly notice a bleaching of the absorption especially at low energies. This is attributed mainly to the loss of the carbon-containing side chains, which strongly contribute to the absorption in this range. In addition, acetic acid and water may be lost to ultimately produce inorganic tin oxides (Sn_12_O_18_) (eq 1). The contributions of the different elements and of the different parts of the tin-oxo cage structures to the photoabsorption cross section are visualized in Figure S4.
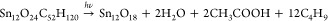
1

Tin is predicted to have an absorption feature at 24.5 eV corresponding to the tin N_4,5_ edge.^50,51^ At the beginning of the exposure, the feature is masked by the strong absorption of carbon, hydrogen, and oxygen. However, as exposure proceeds, more butyl groups dissociate from the tin cage structures, leading to the decreasing contribution of carbon and hydrogen atoms to the absorption of the resist film. Therefore, the tin feature grew relatively and can be detected at longer exposure times.

In the energy range of 42–70 eV, Figure 3B, we can follow the gradual transition from the spectral shape of TinOAc toward the spectrum of tin oxides over the exposure time. The predicted TinOAc absorption spectrum reaches almost a plateau between 45 and 58 eV. Then it follows a decreasing trend for values higher than 58 eV. The turning point of 58 eV in Figure 3B comes from the peak in the cross section of the photoionization of the Sn(4d) electrons near this energy.^52^ This feature becomes more pronounced with increased exposure time as the tin element contribution to the absorption spectrum of the resists grows with exposure due to the outgassing of carbon-containing materials.

### Estimation of Conversion

In Figure 3, we plot the experimental absorption spectra at the beginning (*A*_0_^obs^) and end (*A*_∞_^obs^) of the exposure time, together with the predicted spectra (*A*_∞_^pred^) at the end of the exposure, corresponding to full conversion to Sn_12_O_18_ with the same molecular surface density as the original resist film. The surface number density is defined as *S* = ρ*z*/*M* (mol cm^–2^) in which ρ is the density (typically ∼1.9 g cm^–3^),^53−55^*z* the film thickness and *M* the molecular weight. The predicted spectrum at the maximum conversion (*A*_f_^pred^) is calculated using eq 3 in which *c* is the converted fraction. Using this linear interpolation between the spectra of TinOAc and of Sn_12_O_18_ is an approximation because the butyl groups, water, and acetic acid are not likely to be lost perfectly synchronously, but it gives a useful measure of the overall conversion.
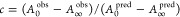
2
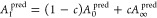
3Using 2 we find that in the low energy region the observed conversion is 69% of the expected maximum. In the high-energy range, the ultimate conversion is 56%. We would expect the maximum conversion to be similar in both energy ranges, although not necessarily identical. The more important observation is that even after prolonged exposure, the organic groups are not fully eliminated from the thin films.

In the photolithography field, the bleaching of a photoresist is characterized by the so-called Dill parameters that describe the bleachable and nonbleachable contributions (*A*_Dill_ and *B*_Dill_, respectively) to the absorption coefficient α = *A*_Dill_ + *B*_Dill_ at the exposure wavelength.^9,56−58^ In the present case, the sample is excited with a broadband of XUV radiation, and the Dill parameters are derived for different wavelengths within that band. Because the main chemical changes (butyl group loss) that cause bleaching are the same at any photon energy above the ionization threshold, *A*_Dill_ and *B*_Dill_ do not depend on the excitation energy but mainly on the probe wavelength, which reflects the absorption spectrum. Figure 3 shows the bleachable and nonbleachable fractions of the absorption as the red and gray areas, respectively. Plots of the absorption coefficient α and Dill parameters obtained using eqs 4 and 5 are shown as a function of energy in Figure S5 (Supporting Information).
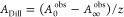
4
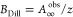
5The values of *A*_Dill_ are slightly higher than those reported for TinOH for the energy range of 25–40 eV, due to higher numbers of oxygen and carbon in TinOAc than in TinOH.^39^

### Quantum Yield

**Figure 4:**
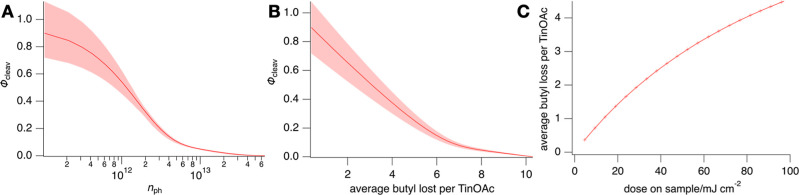
Quantum yields of butyl loss in TinOAc thin films irradiated with photons in the range 22–42 eV and detected using the absorption changes at the 12 HHG peaks in the range 21.7–38.6 eV (eq 9). (A) The horizontal axis shows the cumulative number of photons absorbed. The shaded area corresponds to the range of values of the quantum yield evaluated at different harmonic orders, with details in the text. (B) Average quantum yield vs average number of butyl groups lost per cage. The shaded area represents the statistical error from the 12 different harmonic orders used but does not include the uncertainty in the irradiation dose. (C) Average number of butyl groups lost per TinOAc molecule as a function of the cumulative dose on the sample.

Knowing the absorbance and the dose impinging on the sample in each time interval during the exposure, we can calculate the number of photons absorbed in each time interval. The decrease in absorbance is related to the loss of volatile molecules. Because we know the thickness of the sample and the irradiated area and can estimate the density of the film, we can convert the absorbance change to the number of butyl groups lost. Since we are most interested in the change at the early stages of conversion, we assume here that the absorbance change Δ*A* is only due to the loss of butyl groups. This leads to eq 6, in which *A*_irr_ is the irradiated area (cm^2^) and σ_bu_ is the cross section (cm^2^ mol^–1^) of one butyl group (C_4_H_9_).
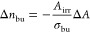
6Plots of the absorbance at each harmonic peak versus the number of photons absorbed are shown in Figure S6 (Supporting Information). The data at each peak energy can be empirically described by using an exponential decay model with two components (eq 7). The fit parameters are given in Table S2 (Supporting Information). Combining eq 6 with eq 8 (the derivative of eq 7) we arrive at the quantum yield of tin-butyl bond cleavage Φ_cleav_ expressed in eq 9. Note that this is an effective quantum yield using broadband irradiation over the range 22–45 eV (Figure S1).
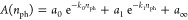
7
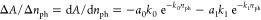
8
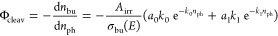
9The quantum yields were evaluated using the absorption changes at each of the different maxima of individual harmonic orders (Figure S6, Supporting Information). There is a variation of the 12 individual values of Φ_cleav_ of ±20% due to random measurement error, but no significant dependence on the photon energy at which it was evaluated (Figure S7). The average value, shown in Figure 4, shows a consistent decrease as the total absorbed dose increases. To quantify this effect in terms of chemical change, we plot in Figure 4B the average quantum yield over all detection energies vs the average number of butyl groups lost. Figure 4C shows the average number of butyl groups lost per TinOAc molecule as a function of the accumulated radiation dose. The observation that a significant butyl loss occurs with a dose of only a few tens of mJ cm^–2^ illustrates why this photoresist is relevant for nanolithography.

### Computational Results

Molecular structures were optimized for the neutral TinOAc, its one-electron reduced and oxidized forms, of the products of one butyl radical loss and of products that have lost two butyl groups. Although for these large molecular systems a high level of theory with quantitative reliability could not be used, the results provide useful guidance for the mechanistic discussion below. Detailed computational results are presented in the Supporting Information. Molecular structures are provided in mol2 format in the data repository associated with the paper. Selected results are discussed below.

## Discussion

**Figure 5:**
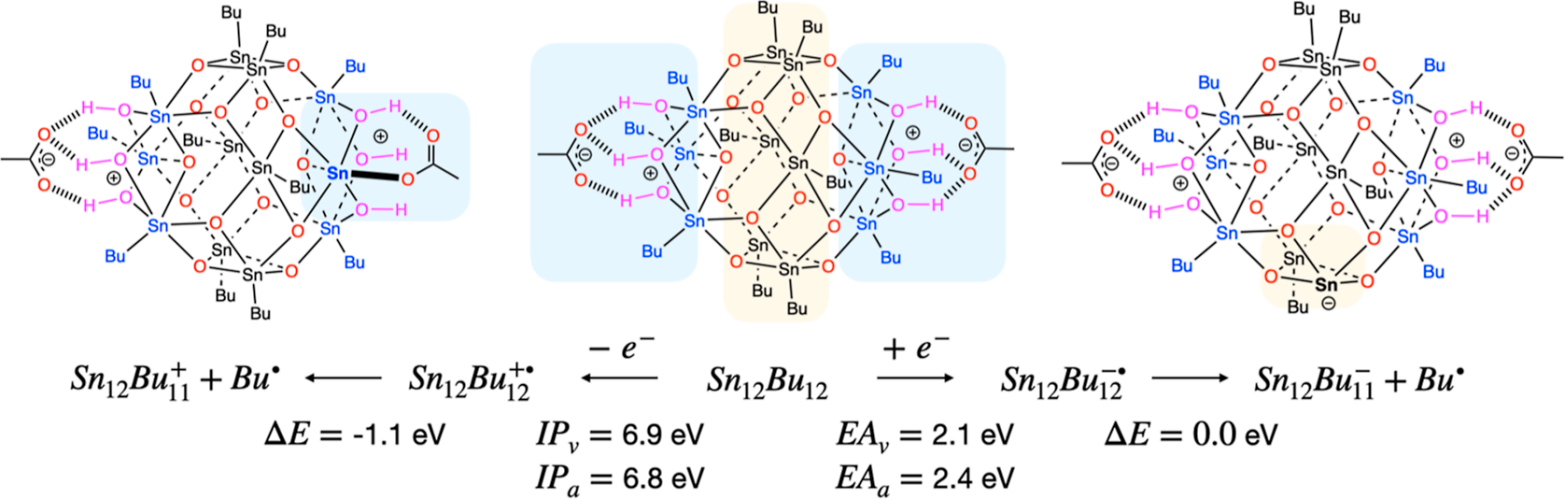
Primary reactions upon electron loss or capture by TinOAc. Computed energies (B3LYP/Def2TZVP(scrf)//Def2SVP) using the PCM model for diethyl ether. IP is the ionization potential, EA electron affinity, subscript *v* denotes vertical, *a* adiabatic. Charges refer to the total system. The highlighted regions in the center structure (Sn_12_Bu_12_, intact TinOAc) are the caps in blue and the belt in ochre. In the reaction products, the highlights indicate the part of the molecule where the chemical change has occurred.

The primary process upon excitation of materials with photons in the XUV range is the emission of a photoelectron. For *h*ν > 30 eV, Sn(4d) electrons (binding energy 29 eV)^59^ can be emitted, although the cross section is negligible at 30 eV, and slowly rises to a maximum near 60 eV according to calculations.^52^ For photon energies >8 eV photoemission from valence electrons is feasible in thin films of tin-oxo cages.^31^ At the EUV energy of 92 eV the yields of valence and Sn(4d) electrons measured using XPS are similar.^59^ Most photoelectrons do not escape from the thin film sample, as their mean free paths are small, at most a few nanometers.^60−64^ In the present work, we focus on the chemical conversion and on the possible reaction mechanisms. The TinOAc molecule consists of a dicationic core structure, with two hydrogen bonded acetate counteranions. In the following, we will use the shorthand notation Sn_12_Bu_*n*_^*x*^ for the degradation products, in which *n* is the number of butyl groups remaining and *x* the total charge on the molecule. TinOAc is a stable molecule up to ∼200 °C.^35^ At the level of theory employed in the present work the Sn–C bond dissociation energy is calculated as 2.4 eV.^35^ Photoionization radically changes the picture: after loss of one electron from the highest occupied molecular orbital (HOMO), which has sigma bonding character,^36,43,65^ an Sn–C bond at one of the caps (six-coordinated Sn atoms) is weakened and elongated. The bond is easily homolytically broken (0.1 eV), but this process is strongly favored because the acetate anion can give up two of its hydrogen bonds and form a new covalent bond with the vacant Sn atom, which is formally +1 charged.^36^ Due to this charge combination, the overall driving force for butyl loss is −1.1 eV. The emitted photoelectron can be trapped by another tin-oxo cage, forming the radical anion Sn_12_Bu_12_^–•^. Also this species is labile, and undergoes Sn–C bond cleavage producing an anionic complex and a butyl radical.^38,65^ In this case, a butyl group is preferentially lost from the central belt of the molecule, where LUMO is predominantly located (Figure 5).

As we have discussed, a single XUV photoionization can initiate two reactions: one via the photoelectron, even when trapped without any excess kinetic energy, and the other in the radical cation, the “hole” that is left behind after photoemission. However, before being trapped on a tin-oxo cage molecule, the photoelectron may excite yet another molecule, and if its energy is high enough, another electron–hole pair may be formed, which again potentially gives rise to two Sn–C bond breaking events. Thus, a single-photon absorption has the potential to initiate more than two reactions. Of course, there are competing processes, such as charge recombination, that reduce the quantum yield. Experimentally, we find that the quantum yield for butyl loss at the beginning of conversion is Φ ≈ 0.9 (Figure 4A,B), which falls in the range of values anticipated. As the conversion proceeds, the quantum yield decreases, and we will consider the reasons for that below.

Breaking of the Sn–C bonds leads to the formation of butyl radicals, which are reactive species. Outgassing products detected upon EUV irradiation (92 eV) are butane, 1-butene and octane, which can be formed by disproportionation or combination of butyl radicals in the gas phase. It is also possible that the butyl radicals already react inside the thin film. For example, a butyl radical could react with a butyl-Sn unit in the same or another tin-oxo cage molecule, producing octane and a tin-centered radical. It could also abstract a hydrogen from a butyl group, giving butane and a carbon-centered radical that may decompose to butene and a tin-centered radical. Such pathways would lead to multiple Sn–C bond breaking events and enhance the efficiency. More research is needed to investigate these possible routes.

The Sn_12_Bu_11_ anion and cation (Figure 5) are closed shell molecules and are relatively stable. The Sn–C bonds in these compounds have computed bond strengths (2.6 eV in Sn_12_Bu_11_^+^ and 2.4 eV in Sn_12_Bu_11_^–^) similar to those in the starting compound (2.4 eV). It is thus likely that these charged Sn_12_Bu_11_ species remain in the film and are further converted only after a new activation event occurs. In EUV patterning experiments we found that heating the exposed sample in air before development enhances the sensitivity, which indicates that metastable species are left in the film after exposure.^35^

Potentially, a thermal electron transfer from a reduced cage to an oxidized cage could give rise to two neutral Sn_12_Bu_11_ radicals, which would easily lose butyl radicals to form stable closed shell Sn_12_Bu_10_ cages: Sn_12_Bu_11_^+^ + Sn_12_Bu_11_^–^ → 2Sn_12_Bu_11_^•^ → 2Sn_12_Bu_10_ + 2C_4_H_9_^•^.

The calculations predict, however, that the electron transfer step is very unlikely (Δ*E* = 38 kcal/mol).

**Figure 6:**
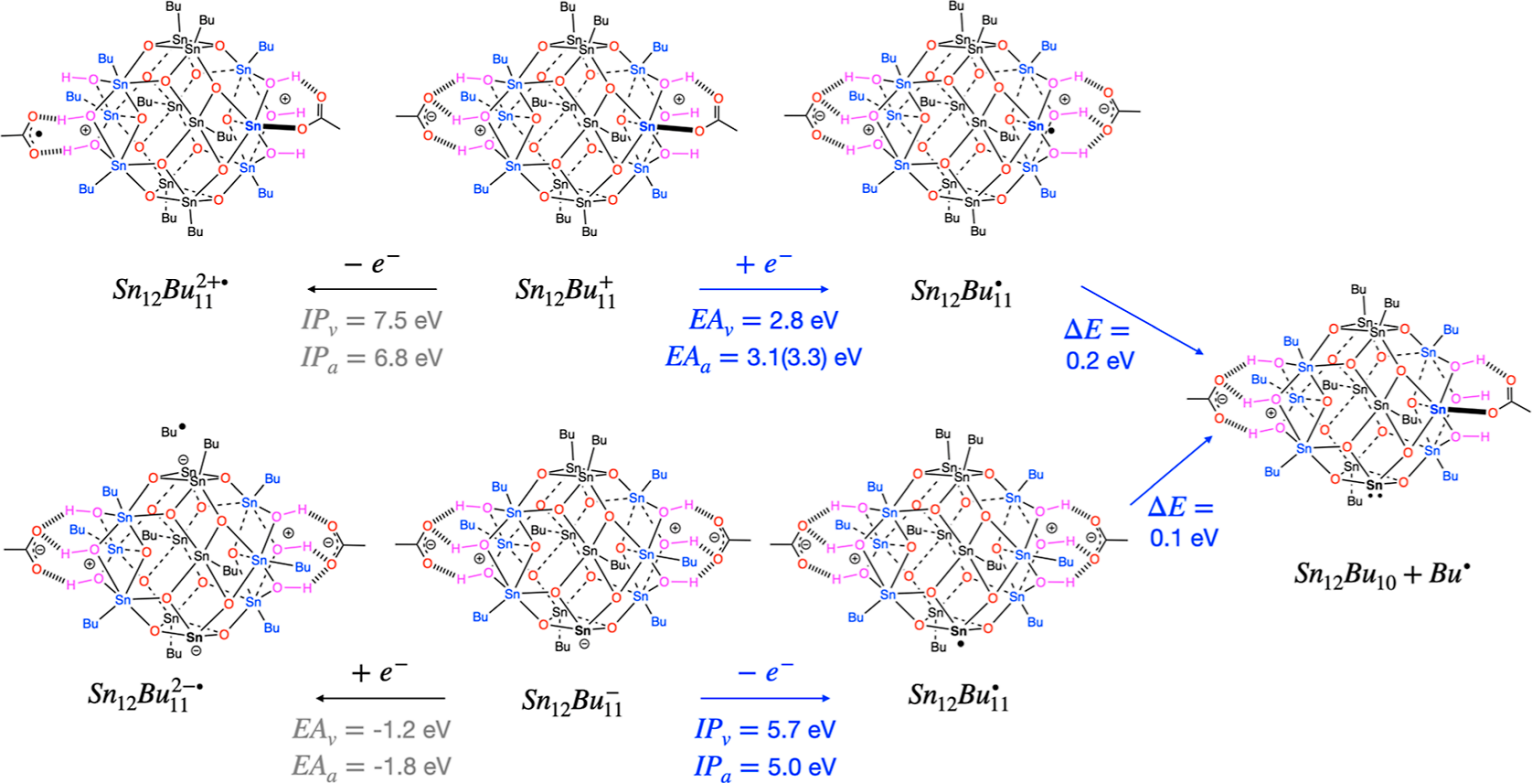
Activation of the Sn_12_Bu_11_ anion and cation via photoionization or electron capture. See the caption of Figure 5 for further details. The two values for *EA*_a_ of Sn_12_Bu_11_^+^ refer to local and global energy minima of the Sn_12_Bu_11_^•^ radical. The more probable pathways are indicated in blue.

Since the Sn_12_Bu_11_ anion and cation are unlikely to decompose rapidly at ambient temperatures via thermally activated reactions, progress of the chemical conversion of the resist requires further activation by photons or by capture of photoelectrons. Ionization of the cation gives a dication radical. Although this is likely to be very reactive, it is a highly oxidizing species that can accept an electron from a neighboring Sn_12_Bu_11_ anion or an unreacted TinOAc molecule, thereby initiating the Sn–C bond breaking in the neighboring molecule. Instead of being activated by photoionization, the Sn_12_Bu_11_ cation can capture an electron that has been generated in a nearby photoionization event, forming an Sn_12_Bu_11_ radical, that readily decomposes to Sn_12_Bu_10_ + Bu^•^ (Figure 6).

**Figure 7:**
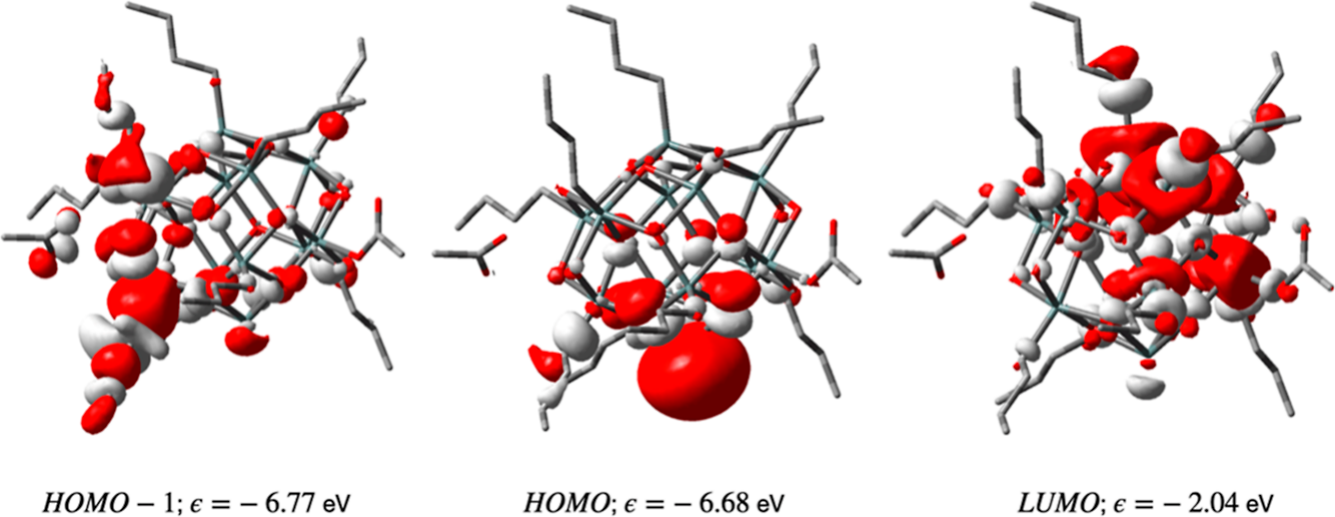
Frontier orbitals of the reaction product (Sn_12_Bu_10_) after the loss of two butyl groups from TinOAc.

The Sn_12_Bu_10_ photoproducts are neutral closed shell species that will not undergo further rapid thermal reactions. The dissociation energy of an Sn–C bond is calculated to be 2.4 eV. The two butyl groups lost as radicals leave two electrons on the cage, and as a result, the HOMO is a lone pair on a tin atom that has been formally reduced from Sn(IV) to Sn(II). The frontier orbitals of the lowest-energy Sn_12_Bu_10_ structure are shown in Figure 7. Ionization of this structure leads to a radical cation (Sn_12_Bu_10_^+^) with the spin density mostly localized on the reduced Sn atom. Upon geometry optimization of the radical cation, spontaneous lengthening of one of the Sn–C bonds does not occur, because the hole is not on a Sn–C σ bond, as it is in the Sn_12_Bu_12_^+•^ radical cation. Although the bond does not show a weakening by elongation, the dissociation energy is Δ*E* = −0.1 eV. The LUMO of the Sn_12_Bu_10_ structure still has the characteristic Sn–C σ* character, but geometry optimization of Sn_12_Bu_10_^–•^ does not lead to breaking of any of the Sn–C bonds. However, a lower energy structure was found, in which the Sn-acetate bond is broken, and the acetate moves back to a hydrogen-bonded orientation. The energy needed to break an Sn–C bond in the anion radical is calculated to be 0.2 eV. Additional computational results on the Sn_12_Bu_10_ and Sn_12_Bu_9_ structures are given in the Supporting Information.

Although the Sn–C bond breaking reactions upon oxidation or reduction of Sn_12_Bu_10_ are thermodynamically feasible, they are energetically less favored than those in Sn_12_Bu_12_. Further loss of butyl radicals leads to more Sn(II) centers, which can act as hole traps. Furthermore, the change in electronic structure compared to Sn_12_Bu_12_ can make the reactions slower, giving more opportunities for competing processes such as electron–hole recombination. In some cases, the acetate counterions participate in the bond breaking reactions. Once both counterions are involved, further conversion will need to take place without their contribution. An additional factor may be the increase in density of the material which hampers the diffusion of organic radicals, facilitating recombination reactions. All these factors may contribute to the decreasing reaction quantum yield with increasing conversion.

## Conclusions

In this study, we have established that the XUV absorption spectrum of thin films of tin-oxo cage TinOAc, as a representative example of EUV photoresist materials, matches well with the spectrum predicted using tabulated cross sections down to energies of ∼22 eV. Prolonged irradiation of resist films with broadband XUV photons leads to photobleaching, which could be described using Dill parameters. For exposure in the range 22–42 eV, an effective quantum yield of the Sn–C bond breaking reaction was determined. At the onset of the reaction, one photon leads to the loss of approximately one butyl group (ϕ = 0.9 ± 0.2). The quantum yield for the loss of the butyl groups gradually decreases to ∼0.15 when half of the butyl groups is lost. The decrease in reactivity is tentatively explained by the change in the molecular electronic structure: the HOMOs of the pristine tin-oxo cage have Sn–C bonding character, and their weakening due to ionization allows the bonds to break, but as more butyl groups are lost the HOMOs are localized lone pairs on Sn atoms that are formally reduced to Sn(II). As the reaction progresses, the material becomes denser, which can also contribute to less efficient chemical conversion, as the higher density may hamper the migration of the butyl radicals, giving more time for recombination reactions to occur.

At the industrially important EUV wavelength of 13.5 nm, the chemical reaction mechanism is not fundamentally different from the one in the XUV range studied in the present work. More secondary electrons will be generated, which can lead to a higher initial quantum yield, but the trend of a strongly decreasing quantum yield with conversion is likely to be the same, which is important for the quantitative modeling of tin-oxo cage EUV photoresists.

In this work, we have gained the first semiquantitative insight into the electronic spectroscopy and chemical reactivity of tin-oxo cages in the XUV range. In the experiments, we used excitation and detection over a range of photon energies simultaneously. More detailed information will be obtainable in the future by monochromatizing the radiation to obtain the reaction quantum yields as a function of the excitation energy.

Further expansion of the knowledge will be needed to fully understand pattern formation in EUV lithography with metal-based resists and to provide a rational basis for optimization of the performance. A pending challenge is to identify experimentally (or with high-level computation) how precisely the decrease in solubility with conversion is related to the structural changes at the molecular level.

## Data Availability

Experimental and computational data are available at 10.21942/uva.24574750.

## Supporting Information Available

Emission spectra of the HHG source, derivation of the absorption spectrum of TinOAc, Dill parameters, absorbance vs dose curves, more detailed description of computational results, and additional results on Sn_12_Bu_10_ and Sn_12_Bu_9_ structures (PDF)

## Abbreviations

HHG: higher-harmonics generation
XUV or EUV: extreme ultraviolet
