# Supplementary material for: XUV Absorption Spectroscopy and Photoconversion of a Tin-Oxo Cage Photoresist
Source: J Phys Chem C Nanomater Interfaces. 2024 Feb 27;128(9):3965–74. doi: 10.1021/acs.jpcc.3c07480 (PMC10926160; doi:10.1021/acs.jpcc.3c07480)
Supplement: Supplementary file 1 — jp3c07480_si_001.pdf [file jp3c07480_si_001.pdf]

Supporting Information

XUV Absorption Spectroscopy and Photoconversion of a Tin-oxo Cage Photoresist

Najmeh Sadegh, Quentin Evrard, Peter M. Kraus, Albert M. Brouwer

Content

Emission spectrum of HHG source	S2
Absorption spectrum of TinOAc	S2
Dill parameters	S3
Quantum yield	S7
Computational results	S8

The experimental data that support the conclusions of this study are available at:
https://doi.org/10.21942/uva.24574750

Emission spectrum of HHG source

The spectra of the irradiance at the sample in the two energy ranges are shown in Figure S1.

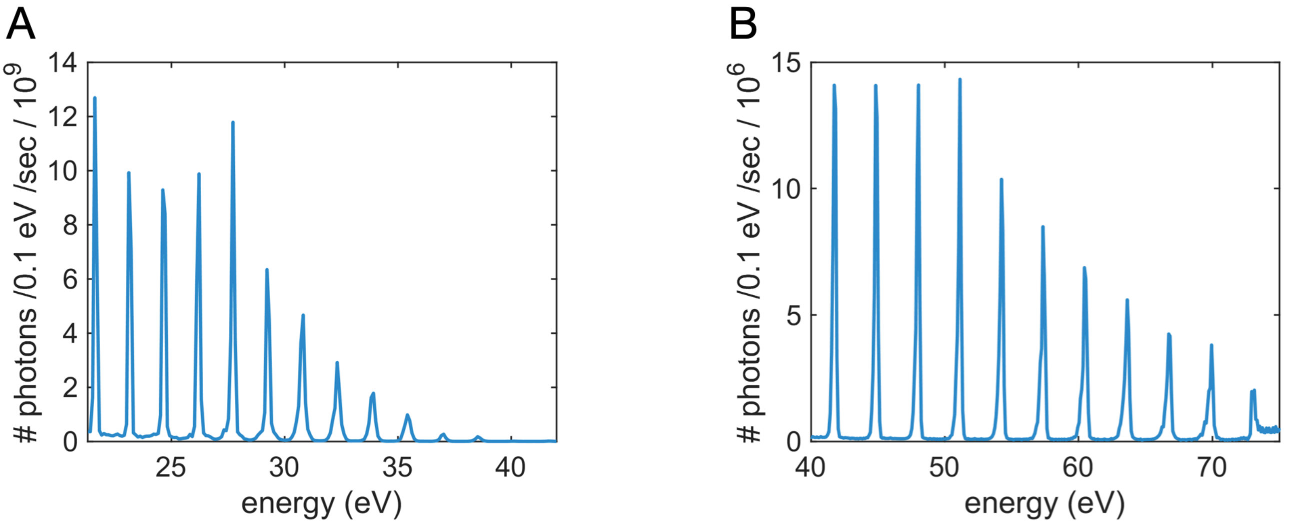

Figure S1. XUV photon flux incident on the sample. a) For argon as the generating medium, and b) for neon.

Absorption spectrum of TinOAc

The individual absorption spectra measured in each of the time intervals are subject to random errors. To correlate the data, we plot the absorbances at each energy vs. exposure dose and fit them with a smooth curve. This allows a consistent extrapolation to $t = 0$ and $t = \infty$. For the low-energy range 22 — 42 eV we use dose on the sample as independent variable. The data are well described using a biexponential decay (Figure S2). For the high-energy range some data points were excluded from the fit because they were obvious outliers, probably due to positioning errors of the sample stage. A single exponential decay proved sufficient. These fits are shown in 2ure S2. Fit parameters are given in Table S1 and Table S2.

Because we do not precisely know the film thickness, we compare the spectra to those predicted using the scattering factors from the CXRO database. For TinOAc, the composition is $Sn_{12}O_{24}C_{52}H_{120}$ (molecular weight M_w = 2554.01 g mol^{-1}). The density ρ of TinOAc films has not been determined experimentally, but from the crystal structures of several butyltin-oxo cages a typical density of 1.9 g cm^{-3} emerges.[1-3] We obtain the predicted absorption spectrum as:

$$A_{pred} = \sigma M_w / \left(\rho z N_A\right) \qquad \text{(S1)}$$

In eq. S1, σ is the cross section (cm^2 mol^{-1}), z the film thickness (cm), N_A Avogadro's number (mol^{-1}). We plot the observed absorbances at the beginning of exposure $A_0(E)$ vs. $\alpha_{pred}(E)$ to find the average thickness z. The predicted spectrum is then $A^0_{pred}(E) = z\alpha_{pred}(E)$. The RMS value of $A^0_{obs}(E)/A^0_{pred}(E)$ is used as an estimate of the relative error in z. The same procedure is applied independently for the data in the two energy ranges. In this way, we find z = 46.2 ± 2.4 nm

for the sample used in the low-energy range and z = 50.5 ± 1.1 nm for the one in the high-energy range.

Dill parameters

The parameters α, A_{Dill} and B_{Dill} as defined in the main text are shown in Figure S4. The experimental data points for both regions are plotted separately. The CXRO-derived spectra that best match the experimental Dill parameters are based on the conversion in the low energy range (69%), which we consider a more reliable result than the conversion in the high energy range because of the larger change in absorbance and better signal-to-noise ratio in the low energy range.

Table S1. Fit parameters for the absorbance change vs dose on sample during exposure in the energy range 20 - 42 eV (Fig. S2).

λ (nm)	A_0	k_0 (cm^2 mJ^{-1})	A_1	k_1 (cm^2 mJ^{-1})	A_∞	$\sigma(A_0)$	$\sigma(k_0)$	$\sigma(A_1)$	$\sigma(k_1)$	$\sigma(A_\infty)$
57.14	0.678	1.44	0.674	12.1	1.378	0.020	0.05	0.026	0.9	0.003
53.43	0.561	1.42	0.646	12.4	1.183	0.013	0.04	0.018	0.7	0.002
50.16	0.456	1.95	0.467	15.6	1.222	0.018	0.08	0.023	1.5	0.002
47.25	0.403	1.65	0.413	14.0	1.135	0.014	0.06	0.019	1.3	0.002
44.66	0.406	1.74	0.454	14.4	1.025	0.015	0.07	0.020	1.3	0.002
42.69	0.316	1.55	0.322	12.0	1.014	0.013	0.07	0.016	1.2	0.002
40.21	0.336	1.35	0.350	12.2	0.899	0.012	0.06	0.017	1.2	0.002
38.28	0.291	1.28	0.305	11.5	0.828	0.012	0.07	0.016	1.2	0.002
36.54	0.274	1.32	0.286	12.6	0.781	0.012	0.08	0.018	1.6	0.002
34.93	0.235	1.26	0.264	12.2	0.734	0.011	0.08	0.016	1.5	0.002
33.48	0.213	1.16	0.242	12.0	0.716	0.012	0.10	0.020	2.0	0.003
32.11	0.189	1.49	0.218	15.0	0.690	0.012	0.12	0.019	2.6	0.002
29.70	0.170	2.36	0.212	37.3	0.665	0.014	0.24	0.039	11.5	0.002

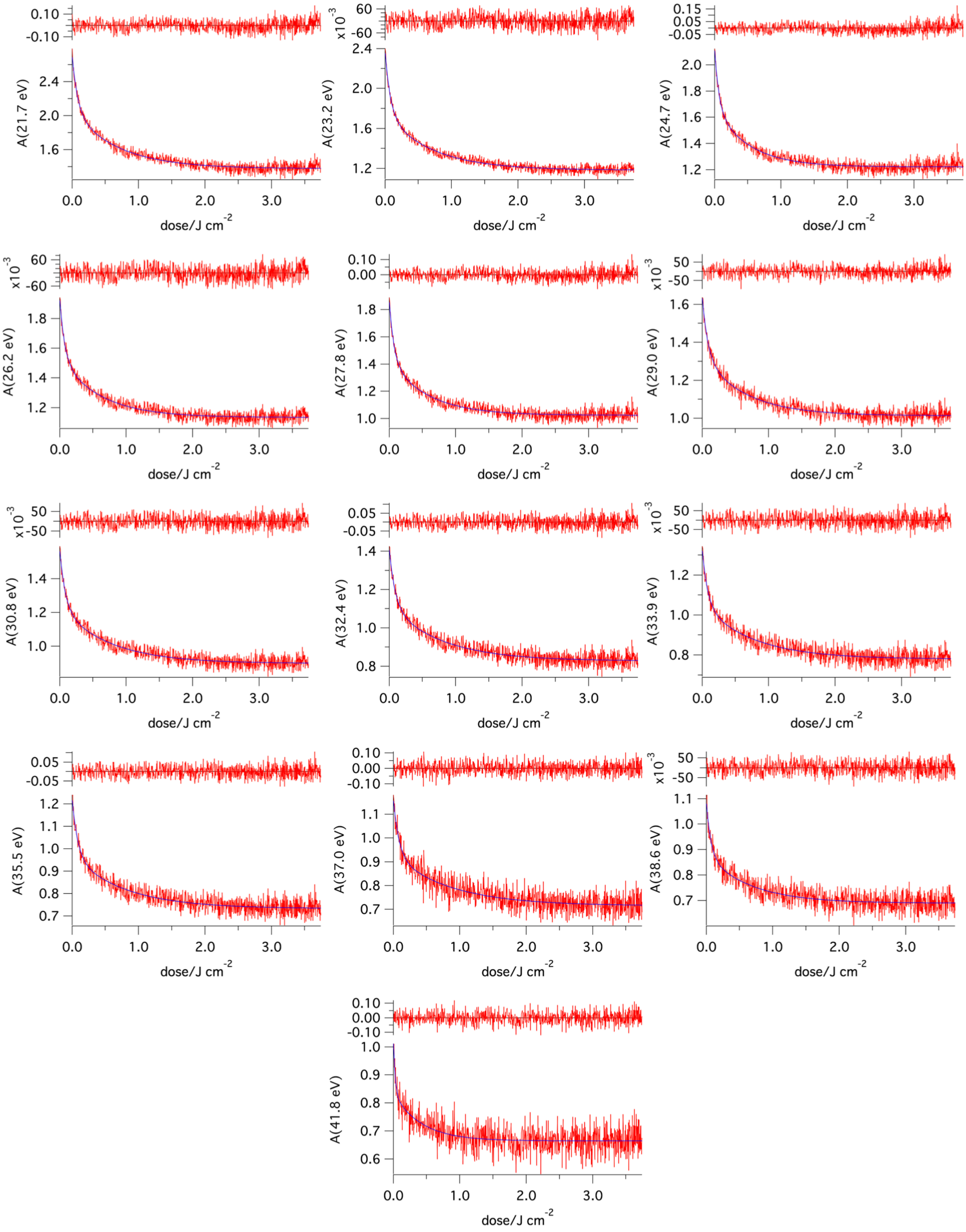

Figure S2. Absorbance vs dose curves for the HHG peaks in the photon energy range 22 — 42 eV. Curves are double-exponential fits to the data: $A(n_{ph}) = A_0 e^{-k_0 d} + A_1 e^{-k_1 d} + A_\infty$ in which d is the accumulated dose (mJ cm-2). The fit parameters are given in Table S1.

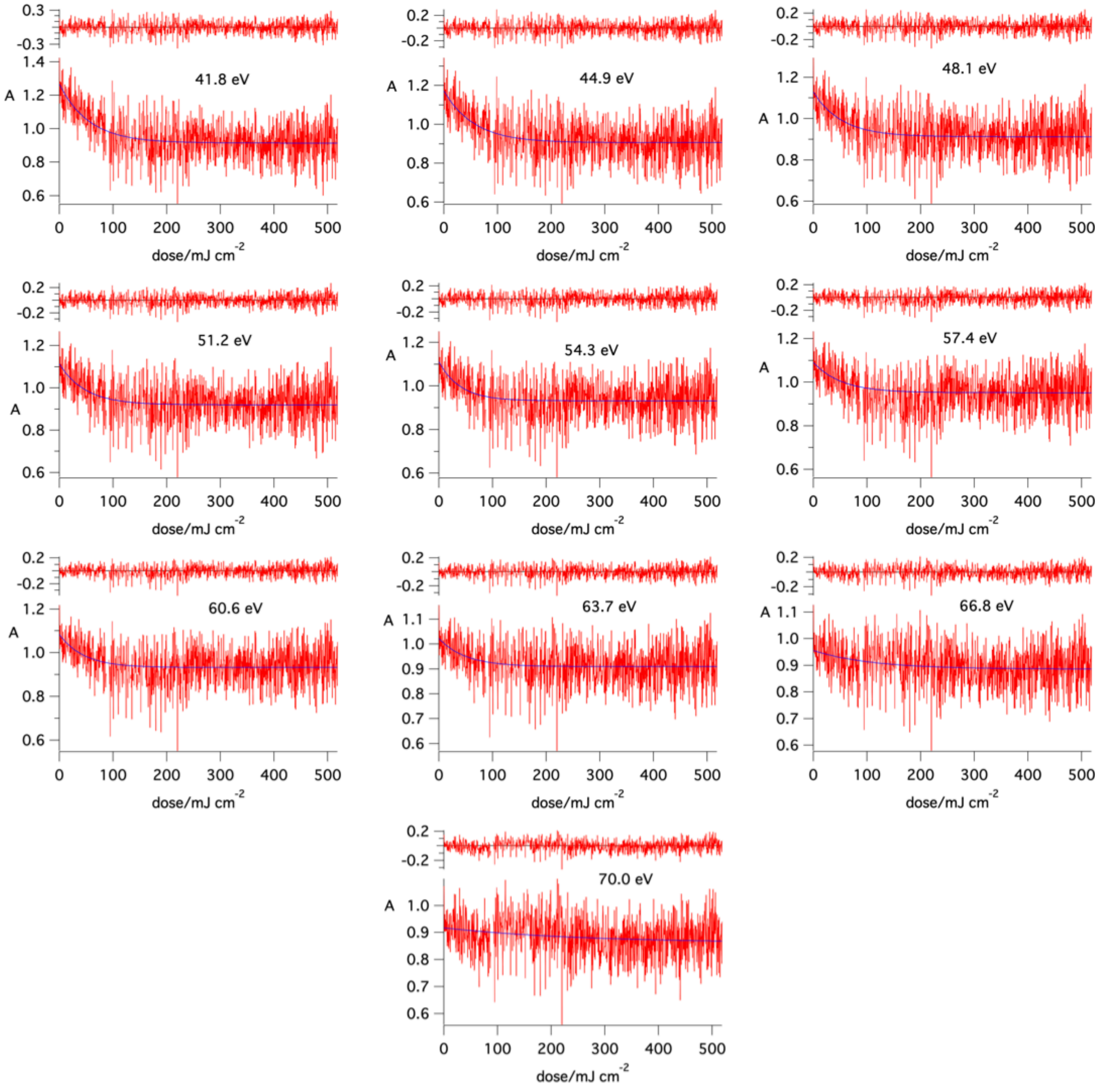

Figure S3. Absorbance vs dose curves for the HHG peaks in the photon energy range 42 — 70 eV. Curves are exponential fits to the data.

Table S2. Fit parameters for the absorbance change vs number of photons absorbed during exposure (Fig. S5)

λ (nm)	A_0	k_0 (photon^{-1})	A_1	k_1 (photon^{-1})	A_∞	$\sigma(A_0)$	$\sigma(k_0)$	$\sigma(A_1)$	$\sigma(k_1)$	$\sigma(A_\infty)$
57.14	0.648	6.041E-13	0.721	7.829E-14	1.377	0.022	2.8E-15	0.028	5.2E-14	0.003
53.43	0.628	6.177E-13	0.596	7.710E-14	1.182	0.015	2.3E-15	0.020	3.8E-14	0.002
50.16	0.446	7.819E-13	0.488	1.054E-13	1.221	0.020	4.3E-15	0.025	8.6E-14	0.002
47.25	0.397	7.021E-13	0.430	8.990E-14	1.134	0.015	3.5E-15	0.020	7.0E-14	0.002
44.66	0.438	7.180E-13	0.433	9.419E-14	1.024	0.017	3.9E-15	0.022	7.1E-14	0.002
42.69	0.308	6.029E-13	0.338	8.436E-14	1.013	0.015	4.0E-15	0.018	6.8E-14	0.002
40.21	0.339	6.110E-13	0.356	7.353E-14	0.898	0.013	3.4E-15	0.018	6.4E-14	0.002
38.28	0.295	5.744E-13	0.308	6.963E-14	0.827	0.013	3.8E-15	0.018	6.8E-14	0.002
36.54	0.278	6.243E-13	0.289	7.146E-14	0.779	0.013	4.2E-15	0.019	8.4E-14	0.002
34.93	0.253	5.842E-13	0.247	6.796E-14	0.733	0.013	4.6E-15	0.018	8.4E-14	0.002
33.48	0.237	5.886E-13	0.224	6.262E-14	0.714	0.014	5.8E-15	0.021	1.0E-13	0.003
32.11	0.213	7.489E-13	0.200	8.097E-14	0.689	0.013	6.5E-15	0.020	1.4E-13	0.002
29.70	0.208	1.831E-12	0.179	1.249E-13	0.665	0.015	1.2E-14	0.041	6.0E-13	0.002

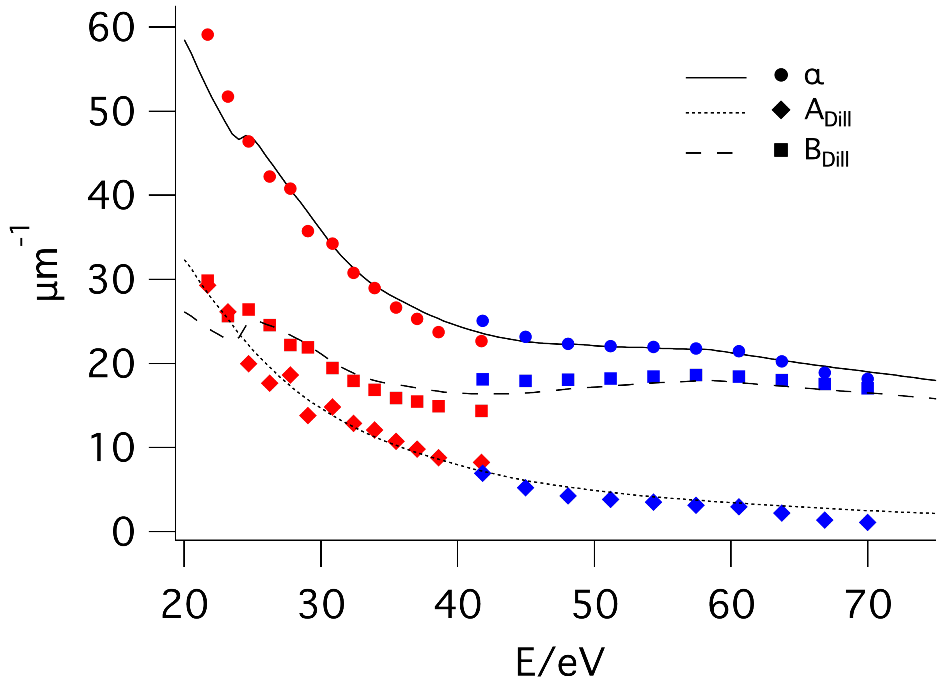

Figure S4. Dill parameters. Red symbols are experimental data in the low energy range, blue symbols experimental data in the high energy range. Solid line: spectrum of TinOAc from CXRO. Dotted line: fitted A_{Dill} using data from low energy range. Dashed line: fitted B_{Dill} using data from low energy range.

Quantum yield

Using the absorption changes as a measure of hydrocarbon loss, and the number of photons absorbed we can estimate the quantum yield of the Sn-C bond cleavage reaction as described in the main text. The curves of absorption versus number of photons absorbed can be fitted to a bi-exponential decay function according to eq. 7 in the main text.The results are shown in Figure S5. Fit parameters are given in Table S2.

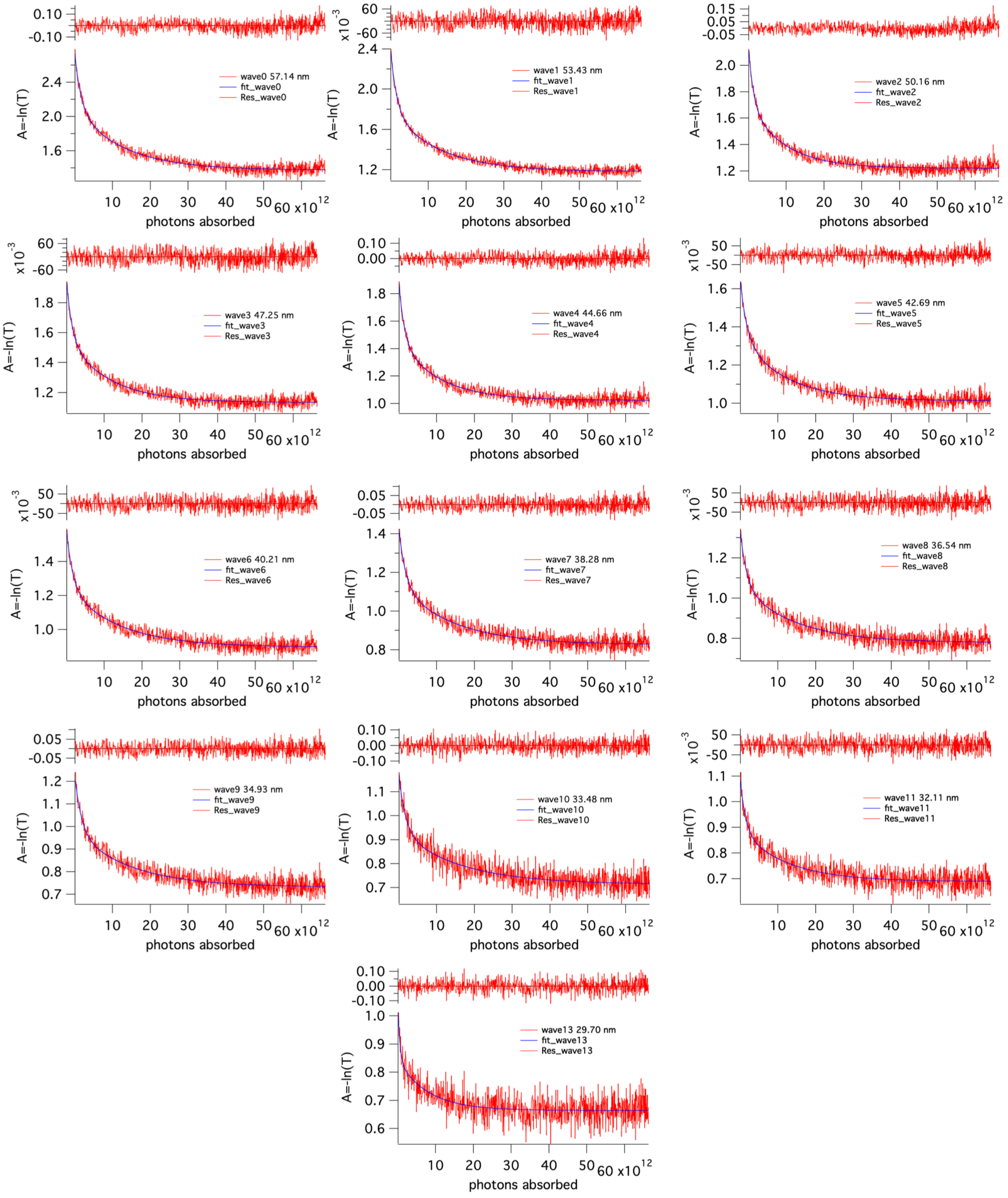

Figure S5. Absorbance at the given wavelengths vs. cumulative number of photons absorbed.

Computational results

Summary data for the most important representative structures are given in Table S4. Molecular structures are provided as mol2 files, with names listed in Table S4. In the text we use the same convention as in the main text with $Sn_{12}Bu_n$ as a shorthand notation for the TinOAc degradation products.

Because of the large conformational space of the molecules we could not explore all possible low-energy isomers. All butyl groups were in extended all-trans conformations but rotations about the Sn-C bonds give many possible conformations. Only the initial structure of TinOAc had c_i symmetry, but all other structures have no element of symmetry. Figure S6 provides a schematic diagram showing the 12 Sn atoms of the tin-oxo cage. We will use this numbering scheme to described where Sn-C bond breaking reactions occur. Also shown is a model of TinOAc from two different viewing angles. The tin-oxo cage can be thought of as composed of a central belt of (Sn-O-Sn-O-) four-membered rings joined at the Sn atoms, and two caps each containing a $(Sn\text{-}OH\text{-})_3$ six-membered ring, with the internal O-atom bonded to all three tin atoms of the cap. Figure 5 in the main text also highlights these three fragments.

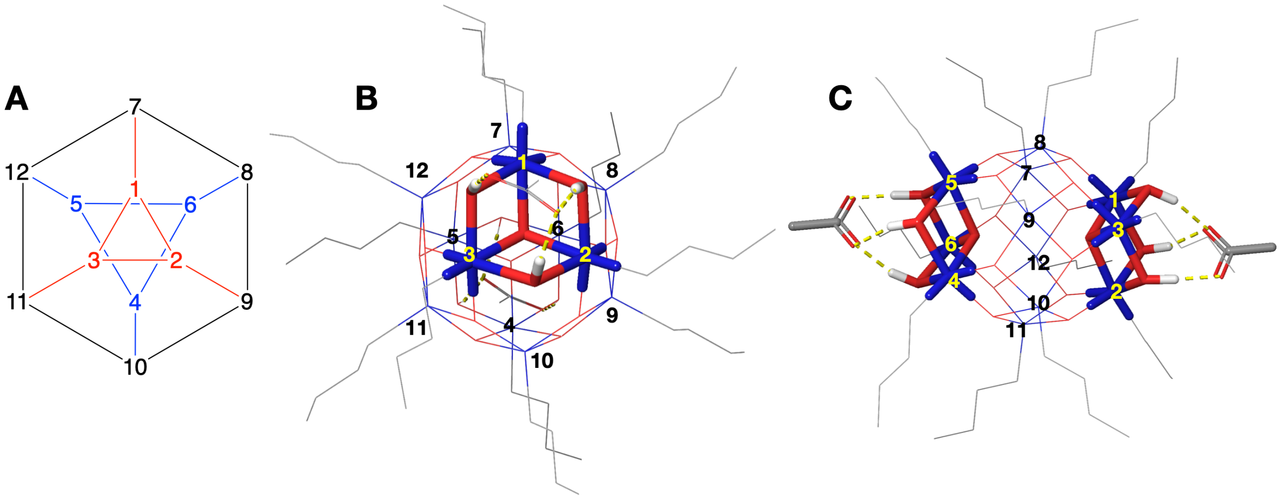

Figure S6. A. Diagram representing the structure of the tin-oxo cage. Only the Sn atoms are shown. The view is along the long axis of the molecule. Each line represents a pair of oxo-bridges between two tin atoms. The triangle 1-2-3 (in red) represents the front cap, with OH bridges, the triangle 4-5-6 the cap in the back. Sn atoms 1, 2 and 3 are bound to an oxygen atom on the inside of the cage, and the same holds for 4, 5, and 6. The 5-coordinated Sn atoms in the belt are displayed in black. Each pair of neighbours is bridged by two O atoms. B. View of TinOAc, in an orientation similar to that in A. The front cap is highlighted using the tube display. C. View along the short axis. Both caps are highlighted, and the hydrogen bonds to the acetate anions are clearly visualized.

Optimization of the radical cation of TinOAc led to a structure with one cap Sn-C bond elongated, as found previously.[4-6] Optimization of the radical anion led to a structure in which a butyl radical is loosely associated with the tin-oxo cage anion.[5]

For the removal of one butyl group, forming $Sn_{12}Bu_{11}$, the main choice is between cleavage from the cap or from the belt. Depending on the charge, different modes are favored: in the neutral

cleavage, a radical is formed preferably on one of the cap Sn-atoms. After one-electron oxidation ($Sn_{12}Bu_{11}^{+}$) bond breaking also occurs at this site. A large energy gain results when the acetate counterion binds with the vacant tin atom. After one-electron reduction, however, cleavage of one of the belt Sn-C bonds is favored. The structures of $Sn_{12}Bu_{11}^{+}$, $Sn_{12}Bu_{11}^{-}$ and the two isomeric radicals $Sn_{12}Bu_{11}^{\bullet}$ are shown in Figure S7.

The figure shows a clearly different bonding arrangement and geometry for the unsubstituted Sn atoms in the cap and in the belt. The Sn atom in the cap is in the middle of the base plane of a square pyramid, of which four of the five oxygen atoms to which it is bonded form the corners. Apparently this is favorable for a radical or for a cation. The Sn atom in the belt has a very different geometry: it is at the top of a square pyramid with four bonds to the oxygens that form the base plane. This site is favored for the lone pair of electrons that is present in the fully reduced Sn(II).

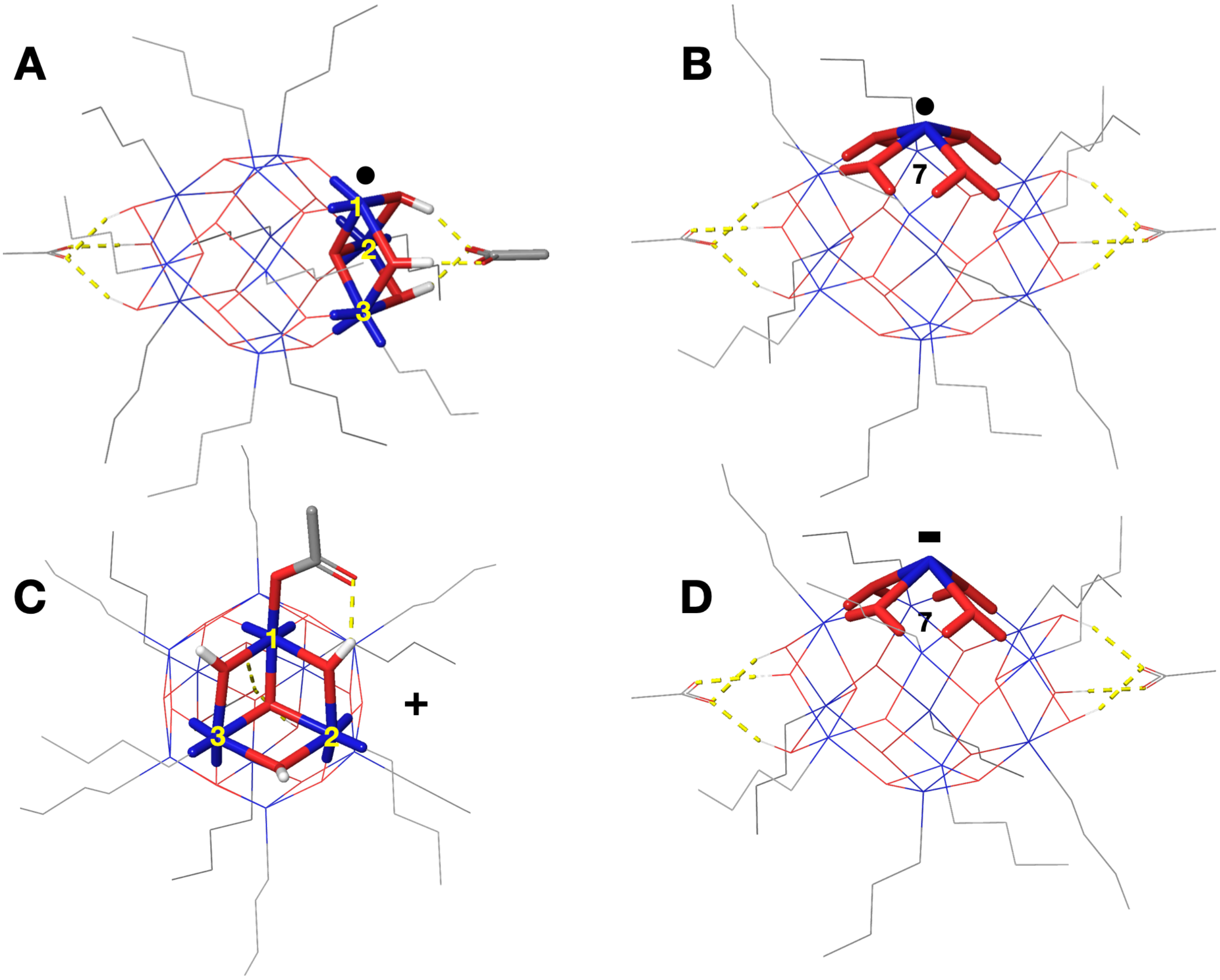

Figure S7. Structures of $Sn_{12}Bu_{11}$ species. A. Radical at cap position (favored). B. radical at belt position (disfavored). C. Cation in which acetate has migrated to the electron-deficient tin atom. D. Anion with a reduced Sn(II) atom in the belt.

Loss of a second butyl group gives rise to $Sn_{12}Bu_{10}$ species. We have explored a number of candidate structures for the neutral, cationic and anionic complexes. To assess the

thermodynamic stability with respect to bond breaking of $Sn_{12}Bu_{10}$ in its three oxidation states we further explored $Sn_{12}Bu_9$ isomers with the corresponding charges.

In all low-energy isomers of neutral $Sn_{12}Bu_{10}$, it is strongly preferred to maintain the Sn-O bond with the acetate. The second free Sn atom carries a lone pair. The calculations show that it is favorable to locate this at an originally 5-coordinated tin site (Sn7). Structures with the lone pair on one of the other 5-coordinated tin atoms are close in energy. A simplified representation of $Sn_{12}Bu_{10}$ is shown in Figure S8.

The vertical ionization potential of $Sn_{12}Bu_{10}$ is $IP_v = 6.5$ eV. Upon geometry optimization a local energy minimum is found with the same substitution pattern ($IP_a = 5.7$). A lower energy isomer of $Sn_{12}Bu_{10}^{+\bullet}$, however, can be found by moving a butyl group from Sn6 to Sn7. The energy difference between $Sn_{12}Bu_{10}$ and the most stable form of $Sn_{12}Bu_{10}^{+\bullet}$ (which we may call global adiabatic IP) is $IP_{a,glob} = 5.5$ eV. We observe that the radical site is preferably located on a cap tin atom (Sn6), with a planar environment, not on a belt tin atom.

Upon one-electron reduction $Sn_{12}Bu_{10}^{-\bullet}$ is obtained. The vertical electron affinity is $EA_v = 2.0$ eV. Geometry relaxation gives $EA_a = 2.6$ eV. In this structure, the bond between Sn1 and the OAc group is broken, and the OAc anion is again hydrogen bonded to the three OH groups. The planar Sn1 is the radical site, the pyramidal Sn7 is the site of the lone pair. A structure with a slightly lower energy (0.3 kcal/mol) was found (lone pair on Sn12 instead of Sn7), but given the accuracy of the calculations this is a negligible difference.

As expected, in neutral $Sn_{12}Bu_{10}$ the third butyl loss is unfavorable ($\Delta E = 2.4$ eV), because the Sn-C bond breaking generates two radicals. Cleavage occurs preferentially on Sn6. As is evident in the structure of $Sn_{12}Bu_9^{\bullet}$ depicted in Figure S7, Sn6 is coordinated by 4 O atoms in a plane and one on the inside of the cage. This local geometry is characteristic of a radical site which is preferably on the cap of the tin-oxo cage.

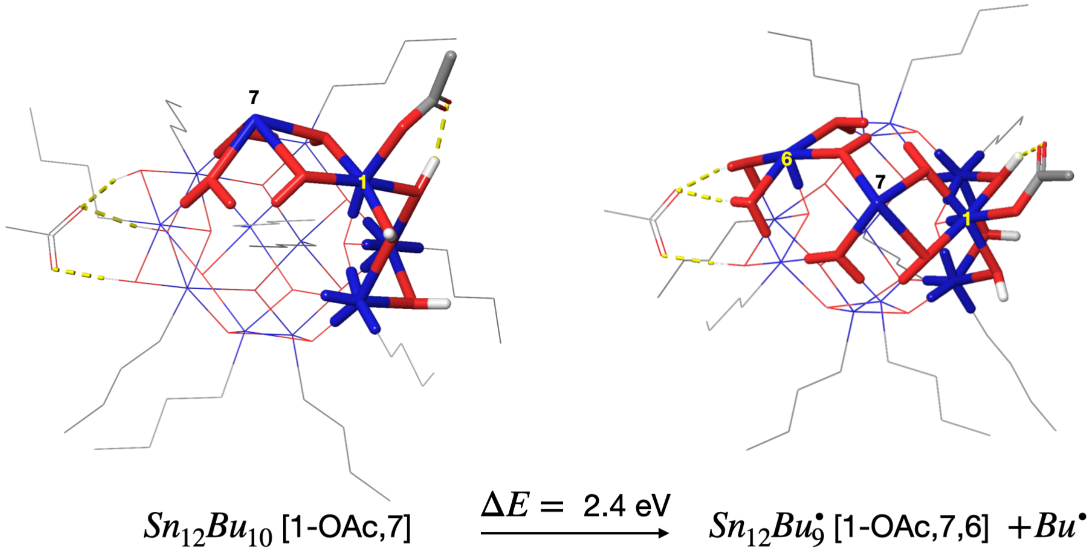

Figure S8. Structure of $Sn_{12}Bu_{10}$ and $Sn_{12}Bu_9$ in neutral oxidation state. The numbers in brackets refer to Figure S6A.

Bond cleavage in the oxidized $Sn_{12}Bu_{10}^{+\cdot}$ is facile. Although the SOMO is mostly localized at the Sn(II) site (Sn7), the Sn-C bond of one of the cap tin atoms is still easily broken: the dissociation energy is $\Delta E =$ -0.1 eV relative to the most stable isomer ($Sn_{12}Bu_9^+$[1-OAc,7,6-OAc]).
In the reduced $Sn_{12}Bu_{10}^{-\cdot}$ the third butyl group is lost from Sn10, giving $Sn_{12}Bu_9^+$[1-OAc,7,10], with a binding energy loss of $\Delta E =$ 0.2 eV.

Table S4. Energies of the most stable isomers that were located for the n-butyltin-oxo cage and its initial reaction products expected to be formed under ionizing radiation. The B3LYP hybrid functional was used throughout.

species	q[a]	S[b]	Def2SVP (optimized)	Def2SVP SCRF // Def2SVP[c]	Def2TZVP// Def2SVP	Def2TZVP SCRF// Def2SVP	structure file name
**$Sn_{12}Bu_{12}$**	0	1	-6429.79842	-6429.81681	-6434.37188	-6434.39147	SnBu12_diA
**$Sn_{12}Bu_{12}^{+\bullet}$**	1	2	-6429.53663	-6429.58221	-6434.08978	-6434.14295	SnBu12_diA_p
**$Sn_{12}Bu_{12}^{-}$**	-1	2	-6429.85692	-6429.90375	-6434.43154	-6434.47858	SnBu12_diA_m
**$Sn_{12}Bu_{11}^{+\bullet}$**	1	1	-6271.88438	-6271.93064	-6276.28624	-6276.33372	SnBu11_diA_p
**$SnBu_{11}^{-}$**	-1	1	-6272.18186	-6272.22959	-6276.58351	-6276.63134	SnBu11_diA_m
**$Sn_{12}Bu_{11}^{\bullet}$[d]**	0	2	-6272.03413	-6272.05306	-6276.43446	-6276.45472	SnBu11_diA_Sn1
**$Sn_{12}Bu_{11}^{\bullet}$[e]**	0	2	-6272.03082	-6272.04940	-6276.42947	-6276.44922	SnBu11_diA_Sn7
**$Sn_{12}Bu_{10}$**	0	1	-6114.35080	-6114.36981	-6118.57913	-6118.59895	SnBu10_diA
**butyl**	0	2	-157.67129	-157.67162	-157.84785	-157.84834	butyl
**butane**	0	1	-158.34285	-158.34257	-158.51984	-158.52003	butane
**1-butene**	0	1	-157.10809	-157.10809	-157.28490	-157.28579	butene

[a] total charge, including 2 OAc^- counterions
[b] spin state: 1 = singlet, 2 = doublet
[c] SCRF refers to the PCM model using diethylether as a low polarity solvent mimicking the amorphous solid state environment
[d] butyl removed from 6-coordinated Sn as in $SnBu_{11}^+$
[e] butyl removed from 5-coordinated Sn as in $SnBu_{11}^-$

REFERENCES

(1) Dakternieks, D.; Zhu, H.; Tiekink, E. R. T.; Colton, R. Synthesis, structure and reactions of $[(BuSn)_{12}O_{14}(OH)_6]Cl_2.2H_2O$: Solution studies using ^{119}Sn NMR and electrospray mass spectrometry. *J. Organomet. Chem.* **1994**, *476*, 33-40.

(2) Banse, F.; Ribot, F.; Toledano, P.; Maquet, J.; Sanchez, C. Hydrolysis of Monobutyltin Trialkoxides: Synthesis and Characterizations of $\{(BuSn)_{12}O_{14}(OH)_6\}(OH)_2$. *Inorg. Chem.* **1995**, *34*, 6371-6379.

(3) Eychenne-Baron, C.; Ribot, F.; Sanchez, C. New synthesis of the nanobuilding block $\{(BuSn)_{12}O_{14}(OH)_6\}^{2+}$and exchange properties of $\{(BuSn)_{12}O_{14}(OH)_6\}(O_3SC_6H_4CH_3)_2$. *J. Organomet. Chem.* **1998**, *567*, 137-142.

(4) Haitjema, J.; Zhang, Y.; Ottosson, N.; Brouwer, A. M. Photoreactions of Tin Oxo Cages, Model EUV Photoresists. J. Photopolym. Sci. Technol. 2017, 30, 99-102.

(5) Ma, J. H.; Needham, C.; Wang, H.; Neureuther, A.; Prendergast, D.; Naulleau, P. Mechanistic Advantages of Organotin Molecular EUV Photoresists. *ACS Appl. Mater. Interfaces* **2022**, *14*, 5514-5524.

(6) Haitjema, J.; Wu, L.; Giuliani, A.; Castellanos, S.; Nahon, L.; Brouwer, A. M. UV and VUV-induced fragmentation of tin-oxo cage ions. *Phys.Chem.Chem.Phys* **2021**, *23*, 20909-20918.
